# The role of teamwork and non-technical skills for improving emergency surgical outcomes: an international perspective

**DOI:** 10.1186/s13037-022-00317-w

**Published:** 2022-02-08

**Authors:** Philip F. Stahel, Lorenzo Cobianchi, Francesca Dal Mas, Simon Paterson-Brown, Boris E. Sakakushev, Christine Nguyen, Gustavo P. Fraga, Steven Yule, Dimitrios Damaskos, Andrew J. Healey, Walter Biffl, Luca Ansaloni, Fausto Catena

**Affiliations:** 1 Department of Specialty Medicine, College of Osteopathic Medicine, Rocky Vista University, 8401 S. Chambers Rd, Parker, CO 80134 USA; 2 The Medical Center of Aurora, 1501 S. Potomac St, Aurora, CO 80012 USA; 3 Department of Clinical, Surgical, Diagnostic and Pediatric Sciences, Policlinico San Matteo, University of Pavia, 27100 Pavia, Italy; 4 Lincoln International Business School, University of Lincoln, Brayford Pool, Lincoln, LN6 7TS Lincolnshire UK; 5 Department of General Surgery, Royal Infirmary, University of Edinburgh, Edinburgh, EH16 4SA UK; 6 First Clinic of General Surgery, Research Institute of the Medical University Plovdiv (RIMU), University Hospital St. George (UMHAT), Plovdiv, 4000 Bulgaria; 7 Department of Surgery, School of Medical Sciences (SMS), University of Campinas (Unicamp), Campinas, SP 13.083-970 Brazil; 8 Division of Trauma and Acute Care Surgery, Scripps Memorial Hospital, La Jolla, CA 92037 USA; 9 Department of General and Emergency Surgery, AUSL Della Romagna, Ospedale Maurizio Bufalini, 47521 Cesena, Italy

**Keywords:** Patient safety in surgery, Non-technical skills, Surgeon-related factors, Teamwork, Emergency general surgery

## Abstract

The assurance of patient safety in emergency general surgery remains challenging due to the patients’ high-risk underlying conditions and the wide variability in emergency surgical care provided around the globe. The authors of this article convened as an expert panel on patient safety in surgery at the 8^th^ International Conference of the World Society of Emergency Surgery (WSES) in Edinburgh, Scotland, on September 7–10, 2021. This review article represents the proceedings from the expert panel discussions at the WSES congress and was designed to provide an international perspective on optimizing teamwork and non-technical skills in emergency general surgery.

## Introduction: the WSES 2021 conference

The World Society of Emergency Surgery (WSES) was founded in 2007 with the mission of promoting research, education, clinical quality and patient safety, interdisciplinary networking, and ultimately the global advancement of emergency surgery [1]. The WSES held its 8^th^ International Conference at the historic McEwan Hall in Edinburgh, Scotland, on September 7–10, 2021. The congress was designed as a “hybrid” meeting with the options of in-person and virtual attendance. The congress was hosted by the University of Edinburgh and sponsored by the Royal College of Surgeons of Edinburgh (RCSEd). The challenges of organizing an international hybrid meeting during the resurgence of the COVID-19 pandemic cannot be understated. In spite of the logistic obstacles, the congress had a record number of submissions for both oral and poster presentations, attracting delegates from 42 countries from all continents. The local organizing committee had the pleasure of welcoming more than 100 delegates in person at the McEwan Hall. The Chair of Behavioural Sciences at the University of Edinburgh has been leading cutting-edge research in the field of non-technical skills, surgical sabermetrics, coaching, global surgery, and space medicine. Furthermore, the MSc in Patient Safety & Clinical Human Factors (www.edinburghsurgeryonline.com/courses), run in partnership with the RCSEd, further added to the distinguished expertise of the patient safety panel in attendance at WSES. This forum allowed for a unique opportunity of international experts to convene in a variety of panels that focused around human factors, non-technical skills, and other pertinent aspects related to surgical patient safety. This review article evolved from an expert panel discussion at the 8^th^ International WSES Congress in Edinburgh and was designed to provide an international consensus opinion on the role of team dynamics and non-technical skills in improving patient safety and patient outcomes in emergency surgery.

## Why our current surgical safety protocols are unsafe

More than 200 million surgeries are performed worldwide each year [2]. Any patient admitted to a hospital to undergo a surgical procedure should rightfully expect to be better off after the intervention than before [3]. However, recent reports reveal that adverse event rates for surgical conditions remain unacceptably high, despite multiple nationwide and global patient safety initiatives over the past decade [4]. These include the “100,000 Lives Campaign” and ensuing “5 Million Lives Campaign” by the Institute for Healthcare Improvement, the “Surgical Care Improvement Project” (SCIP) and “Universal Protocol” implementation by the Joint Commission, and the WHO “Safe Surgery Saves Lives” campaign which rolled out the surgical safety checklist on a global scale [5, 6].

In spite of the multitude of patient safety initiatives designed to protect surgical patients, the current regulatory mandates and safety checklists continue to fall short of protecting surgical patients from suffering preventable harm [7]. Two recent studies from Colorado revealed that “never events” such as wrong-site and wrong-patient surgery continue to occur, albeit at a low incidence, in spite of the availability of modern surgical safety checklists [8, 9]. Contrary to other high-risk industries, patient safety in surgery has historically suffered from a lack of standardized protocols aimed at recognizing and preventing medical errors and surgical complications [10–13]. Ironically, the high standard of regulatory compliance-mandated patient safety protocols in the United States originates from decades of work by lawyers, legislators, and patient advocacy groups, rather than from a physician-driven approach of owning patient safety as a surgical responsibility [4]. A study from the American College of Surgeons’ closed claims database revealed that most surgical complications which lead to malpractice claims do not originate from intraoperative technical errors, but rather from deficiencies in non-technical skills, such as communication breakdown [14]. The success of high-reliability organizations (HROs) is based on high-performing teams, standardized communication, and redundant fail-safe backup options which prevent an error from causing harm [15–18].

## The “team dynamics” experience

The context of emergency and trauma surgery is complicated and characterized by high pressure, stress, and time restraints. Lack of knowledge about the trauma causes, the identity of the patients, and their existing circumstances or conditions can negatively affect surgical decision making, leading to less effective clinical choices. Moreover, the situation may not allow the engagement with the patients or their families in order to understand their treatment preferences or wishes. While shared decision making and co-production dynamics in a multistakeholder scenario stand today among the most relevant pillars of the patient-centric healthcare system, such processes may appear particularly challenging or even not be possible at all [19–21]. Furthermore, time constraints may not allow consulting the clinical guidelines or conferring with specialists for second opinions. In such a scenario, non-technical or soft skills look as relevant as technical abilities, as they allow multidisciplinary team members to work together smoothly [22]. Ensuring surgical patients’ safety and satisfactory clinical outcomes is a priority in emergency surgery. Still, while the surgical community has employed several efforts on the technical side, innovating the surgical techniques and summarizing valuable clinical guidelines, a new approach should emerge. Therefore, as it happens in other fields (for example, sports), team performance and outcomes may also be increased leveraging on more intangible aspects, including non-technical skills and team assembly decisions [23].

Starting from this premise, the “Team Dynamics” initiative [24, 25], endorsed by the WSES, stood as a multidisciplinary research project to investigate the role of non-technical skills [26, 27], knowledge translation dynamics [28, 29], and ethics [30, 31] in emergency and trauma surgery teams. An online questionnaire following the CHERRIES checklist for online medical surveys was conducted from January to February 2021, gathering more than 400 fully-filled questionnaires (response rate > 45%) by surgeons from 72 countries [32]. Results underline the perceived importance of non-technical skills, especially leadership and teamwork. Still, just a few surgeons declare to engage with the patients in share-decision making processes actively. While clinical guidelines and training are widely used among the participants, only half of them report using online tools and electronic medical records. Regarding ethical matters, some interesting results arise. The project investigated the role of surgical consent as a tool to engage and talk to the patient about the clinical options, the possible outcomes, and the pros and cons of the treatment, the perceived importance of the team leader as an ethical leader, and the relevance of ethical training [31, 33]. Interesting enough, team members who declared to belong to a diverse group, meaning which members have different features (in terms of gender, age, education, nationality, values) paid the most significant attention to ethical-related matters.

Starting from the Team Dynamics initiative, some takeaways emerge. Two relevant topics, namely the importance of shared-decision making and the adoption of advanced technological tools to empower surgical decision-making, should be on the agenda of the scientific societies to support the surgical community in ensuring the best possible outcomes for their patients [34]. Moreover, diversity in teams may boost the sensitiveness towards patients’ engagement and shared decision making. Therefore, team assembly decisions may represent a valuable strategy to be pursued by those institutions more engaged towards a patient-centric philosophy.

## Human factors in surgery

### Surgical systems

“Human factors” is the science of human behavior and action at work, with the dual aim of enhancing performance and promoting wellbeing. It is a multidisciplinary science, incorporating fields as diverse as engineering, psychology and anatomy, and has particular relevance to surgery [35]. The operating room is a specific type of complex work environment, named a socio-technical system in human factors language [36–38]. These types of systems including petrochemical, transportation, space exploration, and financial technology, comprise humans, technology and processes that all work together in synchrony – often with variable resources, time pressure, inherent risk, and a multi-professional workforce. The “Chartered Institute for Ergonomics and Human Factors” (CIEHF) is the professional body for human factors specialists in the UK and it recently produced a white paper on *Human Factors for Health & Social Care* [39]. This sets out broad principles for delivering a patient safety strategy with three aims:

- Human Factors in healthcare should be systems-focused,
- Improvements in performance are predominantly design-driven,
- There should be an emphasis on improving the wellbeing of patients and staff.

**Fig. 1:**
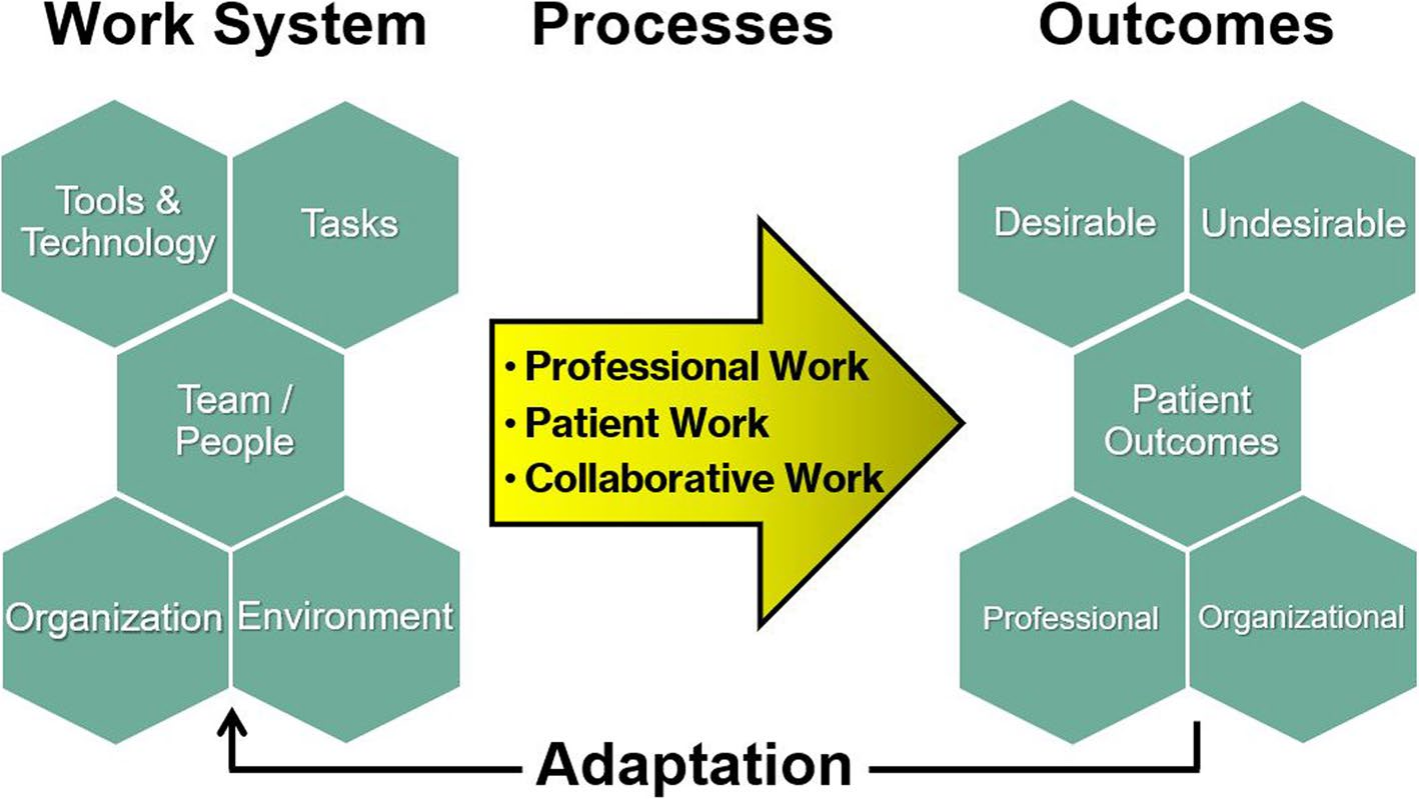
Schematic depiction of the “Systems Engineering Initiative for Patient Safety” (SEIPS) model 2.0. Modified from [40]

Developing systems which support surgical teams to have long and successful working lives, and achieve their full potential has a huge upside for both clinicians and patients. One prominent model used to understand these systems factors in surgery is the “Systems Engineering Initiative for Patient Safety” (SEIPS) model 2.0 [40]. SEIPS 2.0 is a framework for evaluating the complex and dynamic *systems and processes* within healthcare using human factors concepts. Figure 1 depicts the SEIPS 2.0 model which outlines the “Work System” that encompasses five interconnected elements: person, tasks, tools and technologies, the physical environment and organizational conditions. These five interacting elements in turn influence care and other connected processes which in turn impact on patient, staff and organizational outcomes.

The individual person at the centre of the work system could be any healthcare provider or team performing patient care related tasks or a patient receiving care or their family and support system. System design must take into account person characteristics (including age, competence, preferences, ability to manage health information and wellbeing) as well as collective-level characteristics such as team cohesiveness or consistency of knowledge. Human Factors models such as SEIPS allow objective and holistic evaluation of how outcomes are achieved, reduces focus on one particular problem or area to ‘blame’, and can help target areas for improvement.

### Non-Technical Skills

**Fig. 2:**
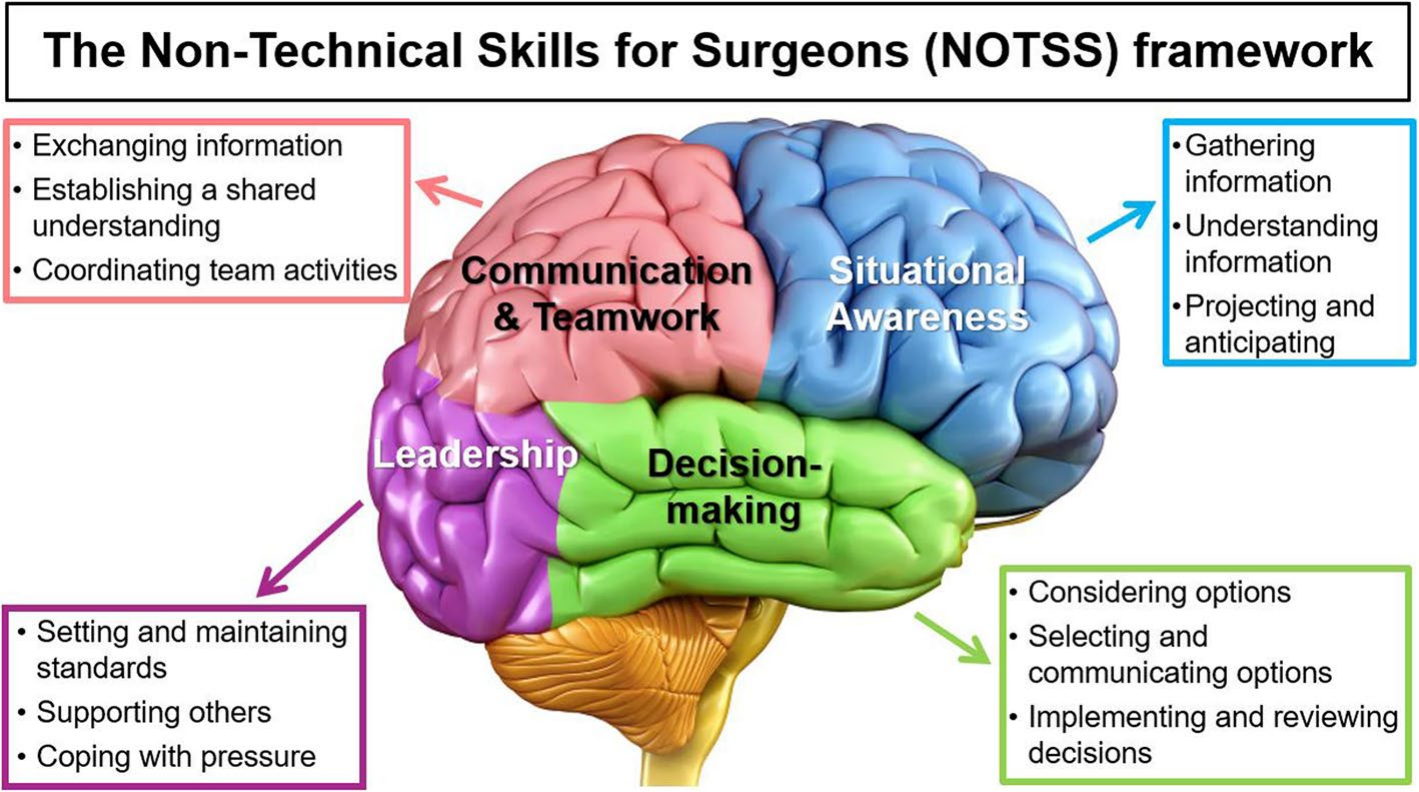
The “Non-Technical Skills for Surgeons” (NOTSS) framework. Modified from [42]

An important subset of Human Factors is called “Non-Technical Skills” (NTS). The NTS are defined as the cognitive and social skills that characterize high performing individuals and teams. They broadly encompass (i) cognitive factors (situation awareness, decision making), (ii) social factors (communication, teamwork, leadership, task management), and (iii) limitations of human performance (dealing with stress, fatigue, rudeness, and burnout). There are several non-technical skills assessment tools available for surgeons and surgical teams. These include the “Non-Technical Skills for Surgeons” (NOTSS) system and the Oxford “Non-Technical Skills (NOTECHS) assessment tool [41–43]. These tools incorporate situational awareness, decision-making, communication skills, teamwork, and leadership (Fig. 2). The NOTSS program teaches structured observation, assessment and improvement in operative behavior and subsequent performance. Many applications in classrooms, clinical, coaching, simulation and virtual learning forums have demonstrated the ability of surgeons to improve these skills. The specific NOTSS definitions provide a breadth of the non-technical skills concept:

- Situational awareness: “Developing and maintaining a dynamic awareness of the situation in the operating theatre, based on assembling data from the environment (patient, team, time, displays, equipment); understanding what they mean, and thinking ahead about what may happen next”.
- Decision-making: “Skills for diagnosing a situation and reaching a judgement in order to choose an appropriate course of action”.
- Team communication: “Skills required for working in a team context to ensure that the team has an acceptable shared picture of the situation and can complete tasks effectively”.
- Leadership: “Leading the team and providing direction, demonstrating high standards of clinical practice and care, and being considerate about the needs of individual team members”.

## Surgeon-related risk factors

### Emergency general surgery and patient safety

**Fig. 3:**
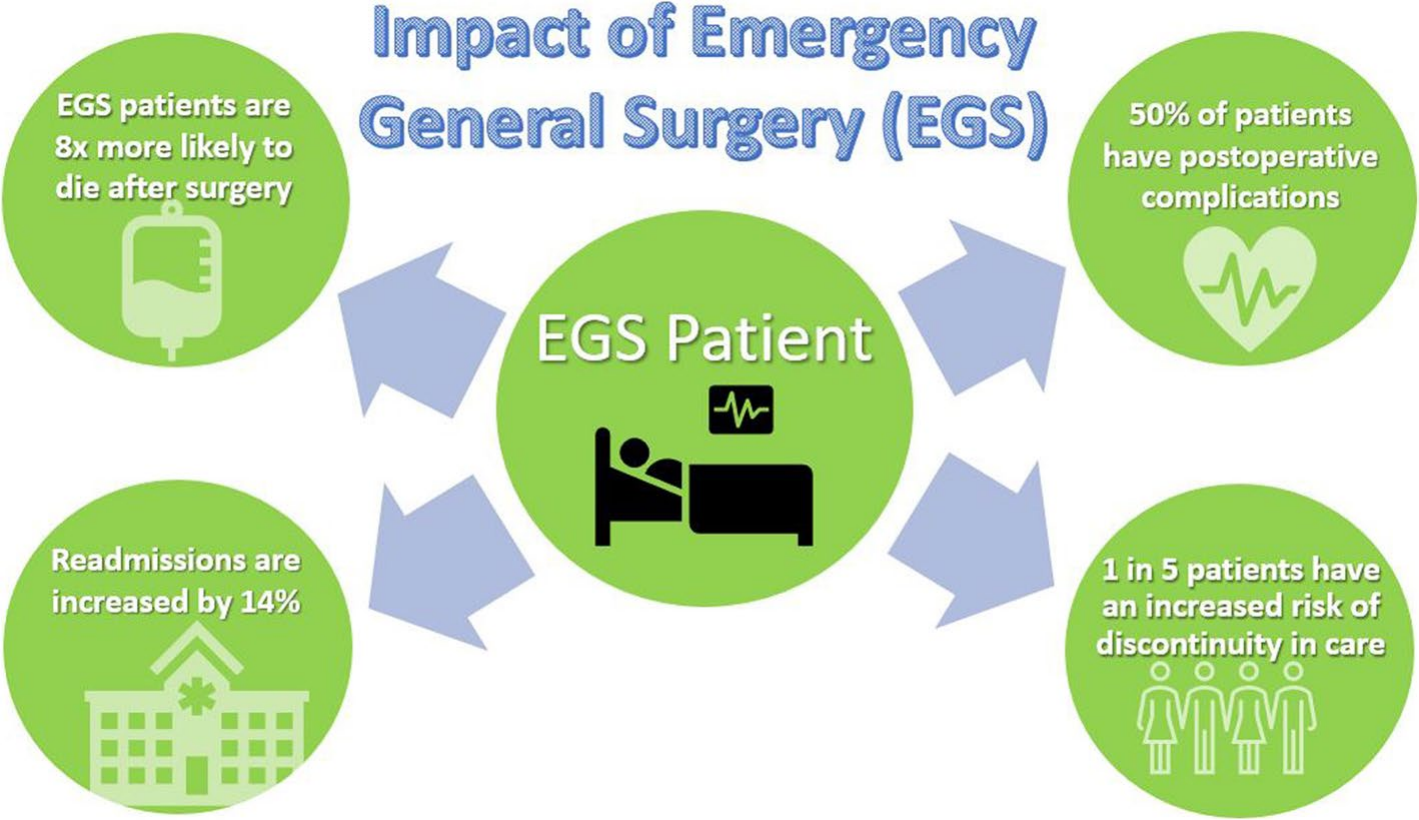
Increased rates of complications and adverse events in patients undergoing emergency general surgery (EGS) compared to elective surgical procedures. Modified from [49]

While patient safety in surgery has received a significant amount of attention in the past decade since the global implementation of the WHO “Surgical Safety Checklist” [6], emergency general surgery (EGS) still represents a neglected entity in the field of patient safety [44–46]. This notion is highly concerning because the rate of postoperative complications is drastically higher in EGS than in elective surgery, and associated with an eightfold increased mortality (Fig. 3) [47]. In addition, the operative burden of EGS is substantial and reflects around one third of surgical volumes in patients admitted to a hospital [48]. Surgeon-related factors in EGS significantly impact patient safety due to a considerable learning curve in surgical training and the complexity and diversity of individual cases with urgent and emergent surgical indications [49]. In spite of improved systems and patient safety protocols, there remains variability in surgical care in EGS due to differing levels of surgeons’ training, experience, and expertise [49].

### What are surgeon-related factors?

Surgeon-related factors for patient safety in EGS are stratified into technical and non-technical skills. While technical skills represent the main focus of surgical residency training, the teaching of non-technical skills has only recently received more global attention [50]. Ironically, most surgical complications originate from poor judgment and deficiencies in non-technical skills, rather than technical mistakes from a “surgical blade gone wrong” [14, 51]. Non-technical skills comprise a wide spectrum of human virtues (“life skills”), including listening and communication skills, situational awareness, decision-making and prioritization, empathy, compassion, emotional intelligence, teamwork, and leadership [49, 51]. These “humanitarian” non-technical skills are mainly pertinent to the perioperative care, including preoperative planning, evaluation of indications and alternative treatment options, shared decision-making with patients, and planning of postoperative care, and may also include intraoperative mindfulness and decision-making skills [51]. In essence, surgeon-related factors can be pragmatically stratified into the pre-, intra-, and postoperative periods in the care of surgical patients. The presence of (or deficiency in) one or more surgeon-related risk factors has been shown to result in potentially preventable surgical complications and adverse patient outcomes [50, 52].

Preoperative surgeon-related factors:

1. Appropriate surgical indication (absolute/relative/not indicated/contraindicated)
2. Shared-decision making for relative indications (alternative treatment options)
3. Surgical planning (imaging/anatomic considerations/surgical approaches)
4. Additional surgical subspecialty consultation/intraoperative availability
5. Hand hygiene and sterile gowning/gloving technique
6. Preoperative antimicrobial prophylaxis (timing/appropriate choice of Abx)

Intraoperative surgeon-related factors:

1. Atraumatic surgical dissection technique and handling of soft tissues
2. Appropriate tension of sutures and selection of suture material
3. Appropriate use of cautery, prevention of intraoperative burn injuries
4. Prevention of tissue ischemia (tension, retractors, tourniquets)
5. Prevention of intraoperative burn injuries (cautery)
6. Prevention of intraoperative contamination (sterile technique, double-gloves, etc.)
7. Intraoperative hemostasis, prevention of intraoperative bleeding and postoperative hematomas
8. Intraoperative rinsing, prevention of tissue dehydration
9. Prevention of prolonged surgical exposure (slow surgeons, teaching cases, etc.)

Postoperative surgeon-related factors:

1. Appropriate postoperative aftercare plan
2. Interdisciplinary team communication for care coordination (mobility orders, thromboembolic prophylaxis, medications, discharge planning, etc.)
3. Monitor, prevent, recognize and mitigate postoperative complications and adverse events
4. Assurance of appropriate follow-up and access to care

Perioperative surgeon-related factors:

1. Surgeon leadership and adherence to surgical safety checklists (site marking, preoperative time-out, instrument and lap sponge counts, postoperative debrief, etc.)
2. Physical and mental state of surgeons (burnout, fatigue, sleep deprivation, addiction disorders, etc.)
3. Streamlined proactive communication with ancillary services (anesthesia, postoperative care unit personnel, nurses, intensivists, etc.)

### Managing surgeon-related factors

Current evidence-based strategies are designed to improve non-technical skills and effective team communication and to optimize surgical patient outcomes by decreasing preventable complications through non-technical skill training [13, 49]. The “5 Whys” approach and the Team STEPPS (“Strategies and Tools to Enhance Performance and Patient Safety”) protocol offer modern evidence-based frameworks designed to optimize team communication and patient outcomes [53–56].

**Fig. 4:**
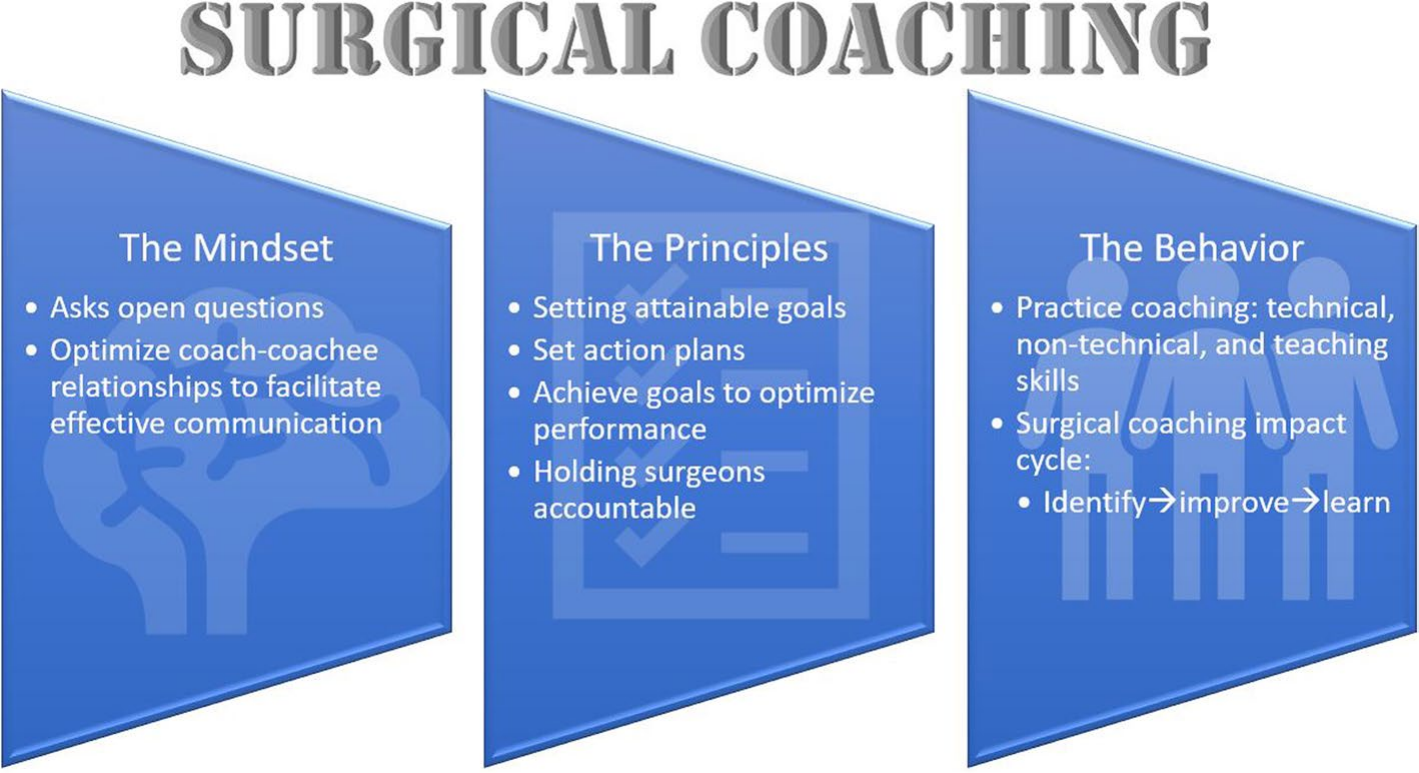
Schematic summary of the content of the “Surgical Coaching for Operative Performance Enhancement” (SCOPE) program. Modified from [57]

Surgical coaching has emerged in recent years as a method to enhance skills and improve patient safety. It borrows techniques from business and sports where coaching is embedded in the culture. There are now a number of coaching programs for surgeons around the world. To highlight one of these; the Surgical Coaching for Operative Performance Enhancement (SCOPE) programme was developed by Ariadne Labs in Boston, USA, and defines coaching as a collaborative partnership between two surgeons to facilitate the pursuit of self-identified goals through collaborative analysis, constructive feedback, and peer learning support (Fig. 4) [57]. The goals of SCOPE are to implement a department-wide surgical coaching program where attending surgeons (“coaches”) coach other peer surgeons (“coachees”) to (i) enable deliberate practice and continuous professional development, and (ii) improve technical and non-technical skill performance. As coaching is still emerging in surgery, there are important questions to be answered such as how to train peer coaches most effectively, what mechanism should be used to match coach and coachee, and which metrics should be used to evaluate surgical coaching [58]. As surgical coaching expands, it is a clear forum to discuss and improve skills that will improve patient safety throughout surgical careers.

A systematic review revealed that poor wellbeing and the presence of burnout among healthcare professionals was associated with a threat to patient safety, including a higher rate of medication errors [59]. In the surgical arena, the risk of preventable patient harm is dramatically increased, due to the complexity in surgical decision-making in conjunction with the intraoperative cognitive workload, flow disruptions, and team performance challenges [50, 60]. The entity of “human performance deficiencies” in surgery represents a combination of errors resulting from cognitive, technical, and team dynamic functions, with cognitive errors representing the most frequent root cause of adverse events and complications in surgery [50, 60]. Cognitive engineering was recently proposed as a successful mitigation strategy designed to decrease surgical complications and improve patient outcomes [60]. The protocol is designed to limit the cognitive demands of humans in “high cognitive workload” environments and thereby reducing the risk of human error. Importantly, cognitive engineering does not replace and *automate* human tasks, but rather *augments* human capabilities by avoiding mundane errors due to distractions and cognitive overload [59]. A classic example in the twenty-first century is reflected by augmented cognition technology in cars with self-driving technology. These modern vehicles do not replace human drivers, but rather monitor the road conditions and alert to unexpected demands whenever driver may be distracted. The fundamental pillars of cognitive engineering pertinent to surgical patient safety are listed in Table 1.

**Table 1:** Cognitive engineering strategies for the prevention and management of errors in surgery. Adopted from [60]

✓ Cognitive aids for high-risk and low-frequency situations	
✓ Surgical time-out	
✓ Sterile cockpit paradigm	
✓ Short breaks	
✓ Team strengthening	
✓ Task shedding	
✓ Intelligent interruption system	
✓ Safety system for device interoperability	
✓ Workload-adaptive systems	

## Opportunities in education and research

The teaching of non-technical skills for medical students and trainees remains a challenge on a global scale. The total number and distribution of medical schools worldwide is not matched with existing physician numbers and distribution. India, Brazil, USA, and China, make up more than one-third of the world’s total number of medical schools [61]. In many countries, only a few selected medical schools have disciplines directed to trauma care and EGS [62]. The simulation assumes growing importance in the field of medical education. Many medical schools work with simulators or even have their own simulation center. Classic emergency training at the hospital or in classrooms are increasingly being replaced by simulation case scenarios. There are many challenges in undergraduate medical education: the need for adjustability and repeatability, high risk of adverse events, limited student’s experience, and students’ fear of the patient. Medical simulation is an effective method of practical education: standardized, objective, adjustable, repeatable, safe, and attractive for the students, avoiding risks to patients and learners. Critical life skills can be controlled and adjusted during simulation scenarios, such as: leadership, collaboration, organization, critical thinking, problem-solving skills, independent learning skills, empathy, tolerance, communication skills, teamwork, and accountability [63, 64]. It is essential to work with NTS concepts with the medical students since the beginning of the course. In the experience at the University of Campinas in Brazil, the skills of medical students have been improved by moving from a predominantly theoretical to a more practical basic life support course (BLS), which included activities on which students were assessed on their ability to teach the principles of BLS to laypeople in the community. Assessing students on their ability to teach improves communication and team work, and can enhance both learning and medical school social accountability [65, 66]. As an extracurricular activity, the medical students in the second year can apply to join the Trauma League, in which they gather with a supervising faculty physician, with the intent to improve their knowledge in the areas of surgery and emergency care; have early contact with environments such as the emergency room, and operating room; observe the surgeons at work (apprenticeship); develop research; organize meetings (regional and national), and work with trauma prevention. It was observed in Brazil the capacity of the Trauma Leagues to bring the students to their actions could be a way to attract new general surgeons and expose them to NTS [67].

The “European Trauma Course” (ETC) brings together different specialists like surgeons, emergency physicians, anaesthetists, intensivists, and others engaged in the initial evaluation of major trauma patients to learn and teach together as a team, including the technical and non-technical skills [68]. The ETC was launched in 2008 during an international conference of the European Resuscitation Council (ERC), composed of members of the European Society of Trauma and Emergency Surgery (ESTES), the European Society of Emergency Medicine (EuSEM), and the European Society of Anaesthesiology (ESA). The ETC course runs over 2.5 days and is predominantly practical based learning assessed on technical, non-technical skills, and team leadership, provided in more than 20 countries around the world [69, 70]. The “Non-Technical Skills for Surgeons” (NOTSS) course has been running for more than a decade in many countries, raising the awareness of the importance of communication in surgical teams, as outlined in detail above.

## Conclusion

The adherence to evidence-based practice protocols and patient safety checklists represents a global trend in EGS [47]. The 19-item WHO “Surgical Safety Checklist” has been implemented worldwide in the past decade and resulted in significant reduction of perioperative morbidity and mortality [6]. However, there remains a wide variability in compliance and adherence to established evidence-based safety checklists [8]. Surgeon ownership and leadership will be required to consistently eradicate preventable complications and adverse events in EGS. The knowledge and proficiency in mastering non-technical skills and endorsing shared decision-making with patients, in conjunction with teaching these non-technical humanistic traits to the next generation of surgeons will hopefully help improve surgical patient outcomes with consistency on a global scale.

## Data Availability

Please contact the author for data requests.
